# Supplementary material for: Annotating high-impact 5′untranslated region variants with the UTRannotator
Source: Bioinformatics. 2020 Dec 14;37(8):1171–3. doi: 10.1093/bioinformatics/btaa783 (PMC8150139; doi:10.1093/bioinformatics/btaa783)
Supplement: btaa783_Supplementary_Data [file btaa783_supplementary_data.zip › Supplementary_Information.pdf]

This is a document illustrating how to use VEP with UTRannotator to identify high-impact 5'UTR variants perturbing upstream open reading frames (uORFs) from scratch.

[Installation - Do you have VEP already installed?]
- [VEP installed]
- [VEP not installed]

[Usage]
- [Basic usage]
  - [Human genetic variants]
    - [Run UTRannotator for genome build GRCh37]
    - [Run UTRannotator for genome build GRCh38]
  - [Genetic variants of other eukaryotic species]
- [Optional usage - evaluate variants on translated uORFs]
  - [Using files of translated uORFs curated from www.sorfs.org]
  - [Using customized files of translated uORFs]

[Output]
- [The detailed annotation for each consequence]
  - [uAUG gained]
  - [uAUG lost]
  - [uSTOP lost]
  - [uSTOP gained]
  - [uFrameShift]

Installation - Do you have VEP already installed?

VEP installed

If you already have VEP installed, the demonstration below is how to install UTRannotator in your system. VEP also provides general instruction on installing and using plugins at https://www.ensembl.org/info/docs/tools/vep/script/vep_plugins.html.

Depending on where you would like to install the plugin UTRannotator, there are two options:

1. Installation of UTRannotator to VEP default path: ~/.vep/Plugins:

```
cd ~/.vep/Plugins
git clone https://github.com/ImperialCardioGenetics/UTRannotator.git
```

2. Installation of UTRannotator to an arbitrary path:

Step 1: Download the UTRannotator GitHub repository https://github.com/ImperialCardioGenetics/UTRannotator/ to your working folder.

Here I am downloading the repository to my working folder `$HOME/example`:

```
cd $HOME/example
git clone https://github.com/ImperialCardioGenetics/UTRannotator.git
```

Step 2: Add the folder UTRannotator to the environment variable `$PERL5LIB` thus UTRannotator could be run with VEP.

To make a permanent change on $PERL5LIB, here I modify the configuration file `~/.bash_profile`:

```
vi ~/.bash_profile
```

Type `i` then add the following line:

```
export PERL5LIB=$PERL5LIB:$HOME/example/UTRannotator
~
~
~
```

Then press esc and type `:wq` to close the file.

Next step is to activate the change by typing `source ~/.bash_profile` in the Terminal.

VEP not installed

If you don't have VEP installed previously, you need to install VEP first by following the instructions here: http://www.ensembl.org/info/docs/tools/vep/script/vep_download.html. One approach, detailed in this link, is to install VEP using a Docker container, which can help alleviate some common issues experienced when installing and first running VEP.

Usage

To Use VEP with UTRannotator, add `--plugin UTRannotator` in VEP command. For command options using VEP, please check here https://www.ensembl.org/info/docs/tools/vep/script/vep_options.html.

All test input files of variants shown in the following examples could be found in our Github repository https://github.com/ImperialCardioGenetics/UTRannotator/tree/master/test.

Basic usage

Human genetic variants

Run UTRannotator for genome build GRCh37

Step 1 - Prepare an input file of variants

For acceptable input formats to use VEP, please check http://www.ensembl.org/info/docs/tools/vep/vep_formats.html#input.

For example, here we have a VCF file `test_grch37.vcf` including a list of variants with human genome build GRCh37 (shown below) we would like to evaluate whether they are 5'UTR uORF-perturbing variants:

```
#CHROM  POS        ID       REF      ALT      QUAL     FILTER   INFO
5       36877039            .        CC       A        .        .        .
8       21988220            .        A        G        .        .        .
1       113498814           .        C        T        .        .        .
11      31828461            .        TAA      T        .        .        .
4       154125682           .        G        GATGC    .        .        .
```

Step 2 - Run UTRannotator (for genome build GRCh37)

Here we use the online database of VEP (no need to download cached file)

```
vep -i test_grch37.vcf \
--database --species homo_sapiens --port 3337 \
--plugin UTRannotator \
--tab --force_overwrite -o test_output_grch37.txt
```

Run UTRannotator for genome build GRCh38

If the uploaded variants are represented using another genome assembly, you can change `--assembly` to specify the genome assembly you would like to use.

```
vep -i test_grch38.vcf \
--database --species homo_sapiens --assembly GRCh38 \
--plugin UTRannotator \
--tab --force_overwrite -o test_output_grch38.txt
```

Genetic variants of other eukaryotic species

UTRannotator can work directly with other eukaryotic species with available genome annotations (https://www.ensembl.org/info/about/species.html) by specifying VEP options `--species` and `--assembly`.

For example, to annotate 5'UTR variants (in the test file `test_mus.vcf)` in mouse *Mus musculus*:

```
vep -i test_mus.vcf \
--database --species mus_musculus --assembly GRCm38 \
--plugin UTRannotator \
--tab --force_overwrite -o test_mus.output
```

Optional usage - evaluate variants on translated uORFs

In UTRannotator, there is an additional option to use a curated list of translated uORFs. With this option, the plugin would check whether the uploaded variants occur in the translated uORFs from that file.

To use this option, simply add the path of translated uORF file after command `--plugin UTRannotator` delimited with comma`,`:

`--plugin UTRannotator, /path/to/uORF_file.txt`

Using files of translated uORFs curated from www.sorfs.org

For translated uORFs in *Homo sapiens*, we have curated a list of uORFs previously identified with ribosome profiling from the online repository of small ORFs (www.sorfs.org): This list is available in our Github repository:

Genome build GRCh37: `uORF_starts_ends_GRCh37_PUBLIC.txt`

Genome build GRCh38: `uORF_starts_ends_GRCh38_PUBLIC.txt`

NB: currently available ribosome profiling datasets are limited in the number of cell types, tissues and experimental conditions they assess meaning that they are likely an incomplete resource of translated uORFs. We include this annotation as increased evidence that a variant may be impactful, but we caution that variants disrupting uORFs without current experimental evidence may also be deleterious.

For example, to evaluate a list of genetic variants in human genome build GRCh38 and whether the perturbing uORFs have translated evidence using the above curated file:

```
vep -i test_grch38.vcf \
--database --species homo_sapiens --assembly GRCh38 \
--plugin UTRannotator,\
$HOME/example/UTRannotator/uORF_starts_ends_GRCh38_PUBLIC.txt \
--tab --force_overwrite -o test_output_grch38.txt
```

Using customized files of translated uORFs

If you have your own curated lists of uORFs, you could convert the information of your lists of uORFs in the following format in a tab-limited file including:

chromosome `CHR`,
start genomic position `START_POS` of the uORF
gene symbol `GENE`,
strand info `STRAND` (either forward or reverse),
UTR type `TYPE` (either five_prime_utr or three_prime_utr),
and end genomic position `STOP_POS` of the uORF

For example, for an uORF of *FOSB*:

CHR	START_POS	GENE	STRAND	TYPE	STOP_POS
19	45971469	FOSB	forward	five_prime_utr	45971714

Output

The output annotation from the plugin includes 5 fields:

For any 5'UTR variants, the plugin will first output the number of existing subtype uORFs in the 5'UTR:

Field 1 - `existing_InFrame_oORFs` : The number of existing inframe overlapping ORFs (inFrame_oORF) already within the 5 prime UTR

Field 2 - `existing_OutOfFrame_oORFs` : The number of existing out-of-frame overlapping ORFs (OutOfFrame_oORF) already within the 5 prime UTR

Field 3 - `existing_uORFs` : The number of existing uORFs with a stop codon within the 5 prime UTR

If this 5'UTR is uORF-perturbing, the plugin will output the consequence and detailed annotation of each consequence. Otherwise it will output `-` :

Field 4 - `five_prime_UTR_variant_annotation` : Output the annotation of a given 5 prime UTR variant.

Field 5 - `five_prime_UTR_variant_consequence` : Output the variant consequences of a given 5 prime UTR variant: uAUG_gained, uAUG_lost, uSTOP_gained, uSTOP_lost, uFrameshift.

If a 5'UTR variant perturbs multiple uORFs, the annotation of each uORF will be concatenated with a logical and symbol `&` for fields five_prime_UTR_variant_consequence and five_prime_UTR_variant_annotation.

The detailed annotation for each consequence

uAUG gained

These are variants that create a new upstream AUG (uAUG) in the 5’UTR, either through changing a single base (i.e. AGG -> ATG) or inserting/deleting bases (i.e. AG -> ATG or ATTG -> ATG).

**Annotations**	**Data type**	**Description**
uAUG_gained_type	String	The type of of 5' UTR ORF created, described by one of the following: uORF(with a stop codon in 5'UTR), inframe_oORF (inframe and overlapping with CDS), OutOfFrame_oORF (out of frame and overlapping with CDS)
uAUG_gained_KozakContext	String	The Kozak context sequence of the gained uAUG
uAUG_gained_KozakStrength	String	The Kozak strength of the gained uAUG, described by one of the following values: Weak, Moderate or Strong.
uAUG_gained_DistanceToCDS	Integer	The distance (number of nucleotides) between the gained uAUG to CDS
uAUG_gained_CapDistanceToStart	Integer	The distance (number of nucleotides) between the gained uAUG to the start of 5'UTR
uAUG_gained_DistanceToSTOP	Integer	The distance (number of nucleotides) between the gained uAUG to STOP codon (scanning through both the 5'UTR and its downstream CDS). If there is no STOP codon found, it would output NA.

uAUG lost

These are variants that disrupt an uAUG that already exists in the 5’UTR, either by a single base change (i.e. ATG -> AGG) or through an insertion/deletion of one or more bases (i.e. ATG -> ATTG or ATG -> AG).

There may or may not be translation from this existing uAUG. We include the ability to annotate these variants with whether or not translation has been detected experimentally from this uAUG. Translation from the AUG is more likely if it has a Strong Kozak consensus.

Annotations	Data type	Description
uAUG_lost_type	String	The type of 5’ UTR ORF lost, described by one of the following: uORF, inframe_oORF or OutOfFrame_oORF
uAUG_lost_KozakContext	String	The Kozak context sequence of the lost uAUG
uAUG_lost_KozakStrength	String	The Kozak strength of the lost uAUG, described by one of the following values: Weak, Moderate or Strong.
uAUG_lost_CapDistanceToStart	String	The distance between the lost uAUG to the start of the 5'UTR
uAUG_lost_DistanceToCDS	Integer	The distance (number of nucleotides) between the lost uAUG to CDS
uAUG_lost_DistanceToSTOP	Integer	The distance (number of nucleotides) between the lost uAUG to the nearest stop codon (scanning through both the 5’UTR and its downstream CDS). Output NA if there is no stop codon.

uAUG_lost_evidence	Boolean	Whether the uORF disrupted by the lost uAUG has any translation evidence. Output NA if no evidence file provided

uSTOP lost

These are variants that disrupt the stop codon of an upstream open reading frame (uORF) that exists within the UTR, either by a single base change (i.e. TAA -> GAA) or through an insertion/deletion of one or more bases (i.e. TAA -> TTAA or TAA -> TA).

There may or may not be translation of this existing uORF. We include the ability to annotate these variants with whether or not translation of the uORF has been detected experimentally. Translation of the uORF is more likely if the start codon (uAUG) has a Strong Kozak consensus.

Annotations	Data type	Description
uSTOP_lost_AltStop	String	Whether there is an alternative stop codon downstream within 5’ UTR
uSTOP_lost_AltStopDistanceToCDS	Integer	The distance between the alternative stop codon (if exists) and CDS. Output NA if there is no alternative stop
uSTOP_lost_KozakContext	String	The Kozak context sequence of the disrupted uORF
uSTOP_lost_KozakStrength	String	The Kozak strength of the disrupted uORF, described by one of the following values: Weak, Moderate or Strong.

uSTOP_lost_FrameWithCDS	String	The frame of the uORF with respect to CDS, described by inFrame or outOfFrame.
uSTOP_lost_evidence	Boolean	Whether the uORF disrupted by the lost stop codon has any translation evidence. Output NA if no evidence file provided.

uSTOP gained

These are variants that add a new stop codon into the sequence of an existing uORF in the same frame as the start codon (uAUG) causing translation of the uORF to end prematurely. As above, the uORF may or may not be translated as we include the ability to annotate these variants with whether or not translation has been detected experimentally.

**Annotations**	**Data type**	**Description**
uSTOP_gained_ref_StartDistanceToCDS	Integer	The distance between the uAUG of the disrupting uORF to CDS
uSTOP_gained_ref_type	String	The type of uORF being disrupted - any of the following: uORF, inframe_oORF,OutOfFrame_oORF
uSTOP_gained_KozakContext	String	The Kozak context sequence of the disrupted uORF
uSTOP_gained_KozakStrength	String	The Kozak strength of the disrupted uORF, described by one of the following values: Weak, Moderate or Strong.

uSTOP_gained_newSTOPDistanceToCDS	String	The distance between the gained uSTOP to the start of the CDS
uSTOP_gained_evidence	Boolean	Whether the disrupted uORF has any translation evidence. Output NA if no evidence file provided.

uFrameShift

These are insertion/deletion variants within the sequence of an existing uORF that alter the reading frame (i.e. they are not a multiple of 3 bases). As above, we include the ability to annotate these variants with whether or not translation of the uORF has been detected experimentally.

**Annotations**	**Data type**	**Description**
uFrameshift_ref_type	String	The type of uORF with the reference allele, described by one of following: uORF, inframe_oORF or OutOfFrame_oORF
uFrameshift_ref_type_length	Integer	The length of uORF with the reference allele. Output NA if there is no stop codon.
uFrameshift_StartDistanceToCDS	Integer	The distance between the start codon of the disrupting uORF and CDS
uFrameshift_alt_type	String	The type of uORF with the alternative allele, described by one of following: uORF, inframe_oORF or OutOfFrame_oORF

uFrameshift_alt_type_length	Integer	The length of uORF with the alt allele. Output NA if there is no stop codon.
uFrameshift_KozakContext	String	The Kozak context sequence of the disrupted uORF
uFrameshift_KozakStrength	String	The Kozak strength of the disrupted uORF, described by one of the following values: Weak, Moderate or Strong.
uFrameshift_evidence	Boolean	Whether the disrupted uORF has any translation evidence. Output NA if no evidence file provided
